# Composite risk and benefit from adjuvant dose-dense chemotherapy in hormone receptor-positive breast cancer

**DOI:** 10.1038/s41523-021-00286-w

**Published:** 2021-06-28

**Authors:** Fabio Puglisi, Lorenzo Gerratana, Matteo Lambertini, Marcello Ceppi, Luca Boni, Filippo Montemurro, Stefania Russo, Claudia Bighin, Michelino De Laurentiis, Mario Giuliano, Giancarlo Bisagni, Antonio Durando, Anna Turletti, Ornella Garrone, Andrea Ardizzoni, Teresa Gamucci, Giuseppe Colantuoni, Adriano Gravina, Sabino De Placido, Francesco Cognetti, Lucia Del Mastro

**Affiliations:** 1 Department of Medicine (DAME), University of Udine, Udine, Italy; 2 Department of Medical Oncology, Centro di Riferimento Oncologico di Aviano (CRO) IRCCS, Aviano (PN), Italy; 3 Department of Internal Medicine and Medical Specialties (DiMI), School of Medicine, University of Genoa, Genova, Italy; 4 Department of Medical Oncology, U.O.C. Clinica di Oncologia Medica, IRCCS Ospedale Policlinico San Martino, Genova, Italy; 5 Clinical Epidemiology Unit, IRCCS Ospedale Policlinico San Martino, Genova, Italy; 6 Day Hospital Oncologico Multidisciplinare, Istituto di Candiolo, FPO-IRCCS, Candiolo, Italy; 7 Department of Oncology, ASU FC University Hospital, Udine, Italy; 8 Oncologia Medica 2, IRCCS Ospedale Policlinico San Martino, Genova, Italy; 9 Department of Breast Oncology, Istituto Nazionale Tumori IRCCS “Fondazione G. Pascale”, Napoli, Italy; 10 Department of Clinical Medicine and Surgery, University of Naples Federico II, Naples, Italy; 11 AUSL-IRCCS Reggio Emilia, Reggio Emilia, Italy; 12 Breast Unit Ospedale S Anna, Citta’ della Salute e della Scienza di Torino, Torino, Italy; 13 Breast Unit, ASL Citta’ di Torino, Torino, Italy; 14 Breast Unit, Department of Medical Oncology AO S. Croce e Carle Ospedale di Insegnamento, Cuneo, Italy; 15 Medical Oncology, S.Orsola University Hospital, Bologna, Italy; 16 Department of Medical Oncology, Sandro Pertini Hospital and S. Eugenio Hospital, ASL Roma2, Roma, Italy; 17 Medical Oncology, Azienda Ospedaliera S Giuseppe Moscati, Avellino, Italy; 18 Clinical Trial Unit, Istituto Nazionale Tumori IRCCS “Fondazione G. Pascale”, Napoli, Italy; 19 Department of Medical Oncology, Istituto Regina Elena per lo Studio e la Cura dei Tumori, Roma, Italy; 20 UO Breast Unit, IRCCS Ospedale Policlinico San Martino, Genova, Italy

**Keywords:** Breast cancer, Outcomes research

## Abstract

The GIM2 phase III trial demonstrated the benefit of dose-dense chemotherapy in node-positive early breast cancer (eBC). To better define the dose-dense effect in the hormone receptor-positive subgroup, we evaluated its benefit through a composite measure of recurrence risk. We conducted an ancillary analysis of the GIM2 trial evaluating the absolute treatment effect through a composite measure of recurrence risk (CPRS) in patients with hormone receptor-positive HER2-negative eBC. CPRS was estimated through Cox proportional hazards models applied to the different clinicopathological features. The treatment effect was compared to the values of CPRS by using the Sub-population Treatment Effect Pattern Plot (STEPP) process. The Disease-Free Survival (DFS)-oriented STEPP analysis showed distinct patterns of relative treatment effect with respect to CPRS. Overall, 5-year DFS differed across CPRS quartiles ranging from 95.2 to 66.4%. Each CPRS quartile was characterized by a different patients’ composition, especially for age, lymph node involvement, tumor size, estrogen and progesterone receptor expression, and Ki-67. A number needed to treat of 154 and 6 was associated with the lowest and the highest CPRS quartile, respectively. Dose-dense adjuvant chemotherapy showed a consistent benefit in node-positive eBC patients with hormone receptor-positive HER2-negative disease, but its effect varied according to CPRS.

## Introduction

Although evidence support dose-dense chemotherapy in early breast cancer (eBC), a clinical practice still varies around the world, and few randomized clinical trials have compared the same drugs and doses in the dose-dense and control arms^1–6^. Furthermore, indirect comparison among trials could also be potentially confounded by noncompletely comparable risk profiles across the patients’ populations enrolled in different studies.

In the clinical decision-making process, careful analysis of clinical and biological characteristics is crucial to identify subgroups of patients with a favorable risk/benefit ratio associated with the use of a dose-dense schedule. Despite the increasing evidence supporting the use of dose-dense adjuvant chemotherapy for patients with high-risk eBC, the association between hormone receptor status and absolute benefit from a dose-dense schedule remains unclear^7,8^.

Previous generation studies often discouraged the use of dose-dense regimens in patients with hormone receptor-positive tumors, restricting the recommendation to the hormone receptor-negative subgroup^7,9^. The CALGB combined analysis of trials 8541, 9344, and 9741, highlighted that dose-dense chemotherapy was particularly effective in patients with hormone receptor-negative eBC, whereas the benefit in patients with hormone receptor-positive disease was smaller and not significantly different from the standard schedule^10^.

The GIM2 randomized phase III trial enrolled patients with node-positive eBC regardless of hormone receptor and HER2 status. After a median follow-up of 7 years, the study demonstrated an improvement both in terms of disease-free survival (DFS) and overall survival (OS) for the dose-dense arm (hazard ratio (HR) 0.77, 95% CI 0.65–0.92, *P* = 0.004 and HR 0.65, 95% CI 0.51–0.84; *P* = 0.001, respectively)^11^. Interestingly, the trial reported a homogeneous benefit among the two hormone receptor subgroups (*P* for interaction of 0.43), with an HR 0.69 for hormone receptor-negative (95% CI 0.48–0.99) and 0.80 for hormone receptor-positive (95% CI 0.65–0.98)^11^.

To further refine the evidence of treatment effect in the hormone receptor-positive subgroup and to better inform selection in node-positive patients between dose-dense versus standard-interval adjuvant chemotherapy, we evaluated treatment benefit according to a quantitative composite measure of recurrence risk based on clinicopathological features.

## Results

### Cohort characteristics and prognostic factors

Characteristics of the 1131 patients in the HER2-negative hormone receptor-positive analysis population according to cohort, as defined by chemotherapy schedule (dose-dense or standard interval), are summarized in Table 1. The median follow-up was 6.4 years (interquartile range 3.8–7.3 years).

**Table 1:** Patients’ characteristics in the total population according to treatment arm.

	Standard *N* (%)	Dose-dense *N* (%)	Total *N* (%)
All patients	586 (51.81)	545 (48.19)	1131
Age (years)			
<35	21 (3.58)	9 (1.65)	30 (2.65)
35–39	43 (7.34)	39 (7.16)	82 (7.25)
40–44	86 (14.68)	71 (13.03)	157 (13.88)
45–49	103 (17.58)	95 (17.43)	198 (17.51)
≥50	333 (56.83)	331 (60.73)	664 (58.71)
Menopausal status			
Premenopausal	297 (50.68)	252 (46.24)	549 (48.54)
Postmenopausal	289 (49.32)	293 (53.76)	582 (51.46)
Nodes			
1–3	361 (61.60)	347 (63.67)	708 (62.60)
≥4	225 (38.40)	198 (36.33)	423 (37.40)
Tumor size (cm)			
≤2	305 (52.05)	299 (54.86)	604 (53.40)
>2	279 (47.61)	244 (44.77)	523 (46.24)
Missing	2 (0.34)	2 (0.37)	4 (0.35)
ER (%)			
<50	97 (16.55)	81 (14.86)	178 (15.74)
≥50	473 (80.72)	456 (83.67)	929 (82.14)
Missing	16 (2.73)	8 (1.47)	24 (2.12)
PGR (%)			
<20	107 (18.26)	103 (18.90)	210 (18.57)
20–49	114 (19.45)	90 (16.51)	204 (18.04)
≥50	319 (54.44)	315 (57.80)	634 (56.06)
Missing	46 (7.85)	37 (6.79)	83 (7.34)
Histological grade			
G1	39 (6.66)	51 (9.36)	90 (7.96)
G2	303 (51.71)	310 (56.88)	613 (54.20)
G3	243 (41.47)	181 (33.21)	424 (37.49)
Missing	1 (0.17)	3 (0.55)	4 (0.35)
KI-67 (%)			
<14	167 (28.50)	198 (36.33)	365 (32.27)
14–19	60 (10.24)	66 (12.11)	126 (11.14)
20–25	113 (19.28)	82 (15.05)	195 (17.24)
≥26	162 (27.65)	132 (24.22)	294 (25.99)
Missing	84 (14.33)	67 (12.29)	151 (13.35)
Endocrine therapy by menopausal status			
LHRH-a	2/0 (0.34/0.00)	1/1 (0.18/0.18)	4 (0.35)
Tamoxifen	86/82 (14.67/13.99)	83/91 (15.22/16.69)	342 (30.23)
LHRH-a + tamoxifen	74/3 (12.62/0.51)	75/7 (13.76/1.28)	159 (14.05)
AI	23/84 (3.92/14.33)	11/99 (2.01/18.16)	217 (19.18)
Tamoxifen + AI	39/94 (6.65/16.04)	35/79 (6.42/14.49)	247 ((21.83)
LHRH-a + Tamoxifen + AI	51/4 (8.70/0.68)	32/2 (5.87/0.36)	89 (7.86)
No endocrine therapy	9/11 (1.53/1.87)	4/5 (0.73/0.91)	29 (2.56)
Missing	13/11 (2.21/1.87)	11/9 (2.01/1.65)	44 (3.89)

**Fig. 1:**
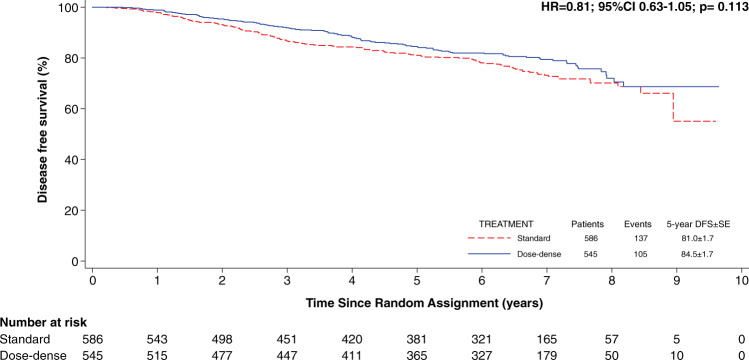
Standard vs dose-dense adjuvant chemotherapy in hormone receptor-positive early breast cancer (eBC) patients. Kaplan-Meier survival plot showing the Disease-Free Survival of patients in the study cohort stratified by treatment.

**Fig. 2:**
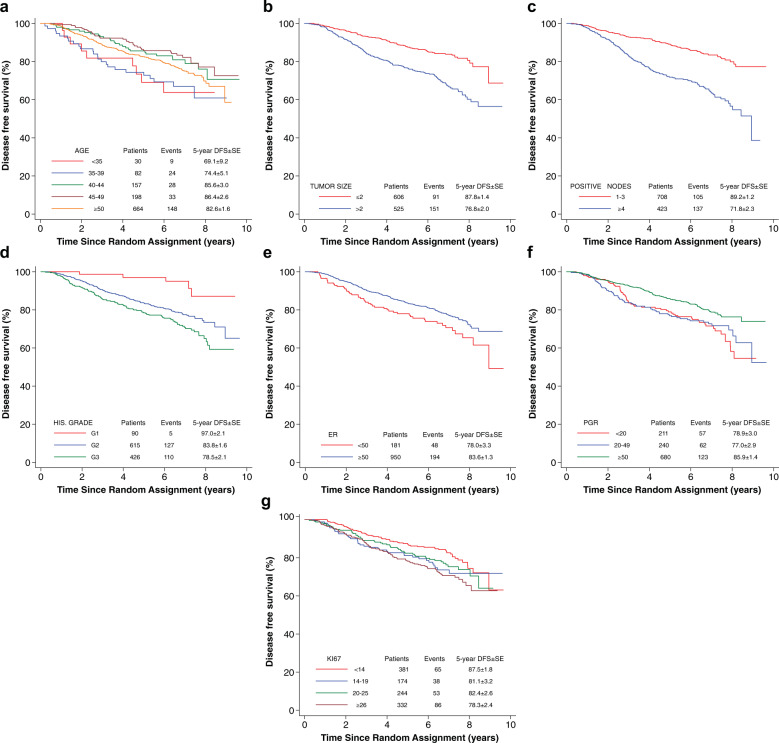
Prognostic factors. Kaplan-Meier plot of DFS independently from treatment assignment in terms of age (**a**), tumor size (**b**), nodal status (**c**), Q9 histological grade (**d**), ER (**e**), PGR (**f**), and Ki-67 (**g**).

The overall 5-year DFS was 84.5% among patients treated with dose-dense chemotherapy versus 81% among patients treated with standard-interval chemotherapy (Fig. 1). The impact of prognostic factors in terms of DFS, independently from treatment assignment, are described in Fig. 2.

The multivariate analysis highlighted that number of positive nodes (≥4 vs 1–3; HR 2.09, 95% CI 1.61–2.71, *P* < 0.001), tumor size (>2 vs ≤2 cm; HR 1.78, 95% CI 1.36–2.34, *P* < 0.001) and histological grade (respectively, G2 vs G1 and G3 vs G1; HR 2.78, 95% CI 1.13–6.80, *P* = 0.026; HR 2.98, 95% CI 1.20–7.40, *P* = 0.018) were the most important prognostic factors in terms of DFS and thus contributed the most to the composite measure of recurrence risk (Table 2).

**Table 2:** Multivariate Cox regression model stratified by treatment for the variables used by composite risk score assessment.

	Log (HR)	HR	95% CI	*P* value
Age				
<35	0.425	1.53	0.72–3.24	0.268
35–39	0.859	2.36	1.39–3.99	0.001
40–44	0.113	1.12	0.68–1.84	0.665
45–49	0.000	1.00	Ref.	
≥50	0.322	1.38	0.94–2.03	0.100
Nodes				
1–3	0.000	1.00	Ref.	
≥4	0.737	2.09	1.61–2.71	<0.001
Tumor size				
≤2	0.000	1.00	Ref.	
>2	0.577	1.78	1.36–2.34	<0.001
ER (%)				
<50	0.262	1.30	0.92–1.82	0.135
≥50	0.000	1.00	Ref.	
PGR (%)				
<20	0.293	1.34	0.96–1.86	0.083
20–49	0.140	1.15	0.82–1.62	0.418
≥50	0.000	1.00	Ref.	
Histological grade				
G1	0.000	1.00	Ref.	
G2	1.022	2.78	1.13–6.80	0.026
G3	1.092	2.98	1.20–7.40	0.018
Ki-67 (%)				
<14	0.000	1.00	Ref.	
14–19	0.166	1.18	0.75–1.85	0.483
20–25	0.068	1.07	0.72–1.58	0.741
≥26	0.095	1.10	0.77–1.57	0.605

The missing values were estimated using the multiple imputation method.

### CPRS quartiles defined subgroups with differential risk and consequent benefit from dose-dense chemotherapy

**Fig. 3:**
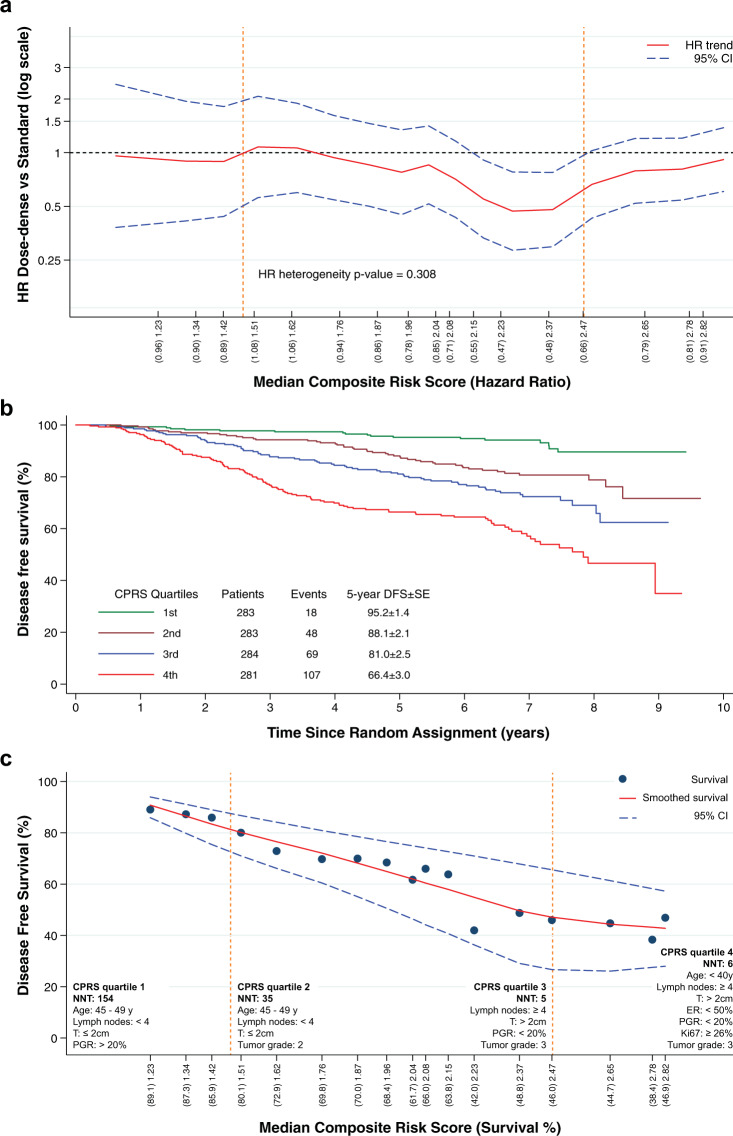
DFS-oriented STEPP analysis. (**a**), CPRS quartiles and prognosis in terms of DFS and 5-year DFS (**b**), disease-free survival according to Median Composite Score (**c**). To smooth the DFS curve and its pointwise confidence interval the LOWESS method with a bandwidth of 0.80 Q10 was applied. Overall, DFS differed widely across CPRS quartiles (**b**), with the number needed to treat (NNT) associated with the lowest CPRS quartile of 154 vs 6 for the highest quartile (**c**). Notably, the first CPRS quartile was characterized by age between 45 and 49 years, less than four lymph nodes involved, tumor size ≤2, and a PGR > 20 (**c**). The fourth CPRS was characterized by an age <40 years, ≥4 lymph nodes involved a tumor size >2, ER < 50, PGR < 20, G3, and a Ki-67 ≥ 26 (**c**). The 25th and the 75th percentiles are represented by the dashed lines.

Table 3 shows the features of the 17 subpopulations identified using the STEPP methodology. As adjacent subpopulations share patients, an overlap in the CPRS range is present. The DFS-oriented STEPP analysis showed different patterns of relative treatment effect with respect to CPRS (Fig. 3a and Table 3). In subpopulations with lower risk scores, the efficacy of dose-dense therapy appears to be absent while for CPRS median values around 2.20 we observed significantly reduced hazard ratios to testify the maximum efficacy of therapy. The hazard ratio increased in patients with a worse anamnestic profile. Overall, DFS differed widely across CPRS quartiles ranging from 95.2% to 66.4% (Fig. 3b). Each CPRS quartile was characterized by a different patients’ composition (Table 4). Considering the observed and expected frequency of each cell estimated from the two-way Table 4, the first CPRS quartile was characterized by age between 45 and 49 years (90 observed vs 51 expected), less than four lymph nodes involved (275 observed vs 177 expected) a tumor size ≤2 (264 observed vs 155 expected) and a PGR > 20 (respectively, 25 observed vs 60 expected and 242 observed vs 170 expected for the PGR 20–49 and ≥50 subgroups). The second CPRS quartile was mainly characterized by age between 40 and 49 years (105 observed vs 91 expected), less than four lymph nodes involved (255 observed vs 177 expected), a tumor size ≤2 (182 observed vs 155 expected), and G2 (169 observed vs 154 expected) (Fig. 3c). Patients included in the third CPRS quartile showed ≥4 lymph nodes involved (132 observed vs 106 expected), a tumor size >2 (141 observed vs 128 expected), a PGR < 20 (69 observed vs 53 expected), and G3 (125 observed vs 107 expected) (Fig. 3c). The fourth CPRS was characterized by an age < 40 years (respectively 12 observed vs 9 expected and 48 observed vs 21 expected for the <35 years and 35–39 years subgroups), ≥4 lymph nodes involved (255 observed vs 105 expected) a tumor size >2 (250 observed vs 127 expected), ER < 50 (70 observed vs 45 expected), PGR < 20 (78 observed vs 52 expected), G3 (155 observed vs 106 expected), and a Ki-67 ≥ 26 (129 observed vs 83 expected) (Fig. 3c). The number needed to treat (NNT) associated with the lowest CPRS quartile was 154, whereas the NNT associated with the highest CPRS was 6 (Fig. 3c). The second and third CPRS quartiles had, respectively, a NTT of 35 and 5.

**Table 3:** Prognosis across composite risk score subpopulations.

Subpopulation	Size	Median CPRS	Minimum CPRS	Maximum CPRS	HR	95% CI
1	302	1.23	0.00	1.49	0.96	0.38–2.42
2	300	1.34	0.70	1.59	0.90	0.41–1.94
3	301	1.42	1.12	1.70	0.89	0.44–1.82
4	302	1.51	1.26	1.82	1.08	0.56–2.07
5	304	1.62	1.36	1.92	1.06	0.60–1.89
6	300	1.76	1.44	2.03	0.94	0.54–1.62
7	306	1.87	1.52	2.08	0.86	0.50–1.46
8	300	1.96	1.65	2.15	0.78	0.45–1.34
9	301	2.04	1.78	2.22	0.85	0.52–1.41
10	302	2.08	1.88	2.34	0.71	0.43–1.16
11	300	2.15	1.97	2.45	0.55	0.33–0.91
12	301	2.23	2.06	2.61	0.47	0.28–0.78
13	300	2.37	2.09	2.75	0.48	0.30–0.77
14	305	2.47	2.16	2.82	0.66	0.43–1.03
15	303	2.65	2.27	2.96	0.79	0.52–1.20
16	300	2.78	2.38	3.29	0.81	0.54–1.21
17	273	2.82	2.49	3.91	0.91	0.61–1.38

*CPRS* composite risk score, *HR* hazard ratio.

Subgroups were defined according to the median composite risk score, interaction *P* value based on hazard ratio estimates: 0.3084.

**Table 4:** Distribution of composite risk score according to the clinicopathological features used for the score assessment.

	CPRS quartiles			
	1st	2nd	3rd	4th
Age				
<35	5.71	25.71	34.29	34.29
35–39	0.00	15.12	29.07	55.81
40–44	32.30	28.57	19.25	19.88
45–49	44.55	29.21	14.36	11.88
≥50	21.48	24.11	28.90	25.50
Nodes				
1–3	38.84	36.02	21.47	3.67
≥4	1.89	6.62	31.21	60.28
Tumor size				
≤2	42.58	29.35	23.06	5.00
>2	3.72	19.77	27.59	48.92
ER (%)				
<50	11.60	22.10	27.62	38.67
≥50	27.58	25.58	24.63	22.21
PGR (%)				
<20	7.58	22.75	32.70	36.97
20–49	10.42	27.08	29.58	32.92
≥50	35.59	25.00	21.18	18.24
Histological grade				
G1	88.89	6.67	3.33	1.11
G2	26.83	27.48	25.37	20.33
G3	8.92	25.35	29.34	36.38
Ki-67				
<14	38.32	24.67	21.00	16.01
14–19	24.14	30.46	23.56	21.84
20–25	21.72	25.00	31.56	21.72
≥26	12.65	22.59	25.90	38.86

**Fig. 4:**
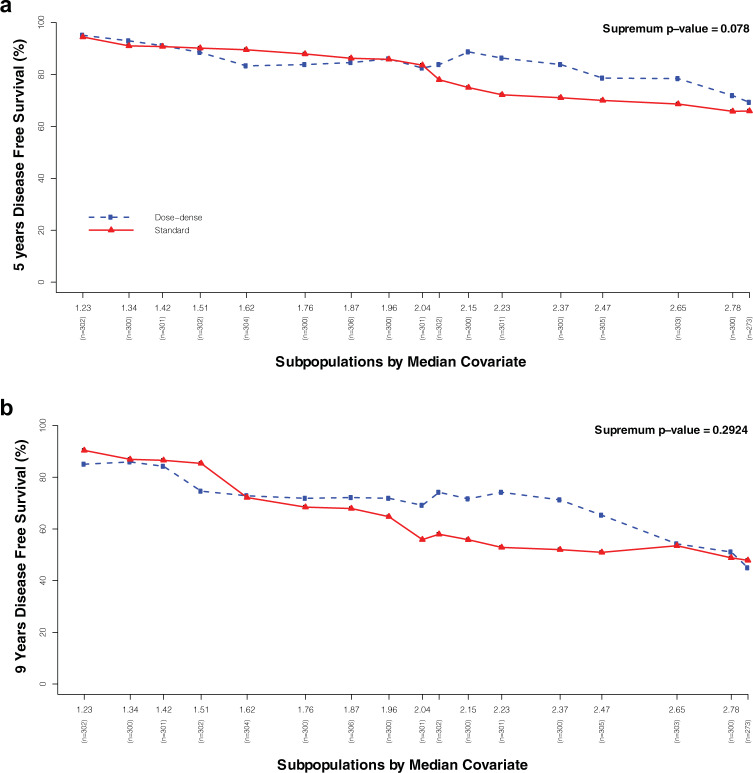
Prognostic impact across treatment types according to CPRS score. Graphical representation of the outcome in terms of 5-year (**a**) and 9-year DFS (**b**).

The CPRS scores’ impact across treatment types was reported in terms of 5-year and 9-year DFS (Fig. 4a, b).

## Discussion

The present analysis conducted within the GIM2 trial assessed the differential benefit of adjuvant dose-dense chemotherapy in hormone receptor-positive HER2-negative eBC patients through a CPRS approach. By combining the prognostic impact of age, nodal involvement, tumor size, level of ER and PGR, histological grade, and Ki-67, 17 different CPRS-based subpopulations were defined, and their HR heterogeneity was represented by STEPP.

The population was then stratified according to CPRS quartiles, suggesting four different risk subgroups with differential benefit from dose-dense adjuvant chemotherapy and characterized by different clinical features. Of note, analyzing the extreme percentiles, it is observed that some variables (e.g., number of lymph nodes <4, tumor size <2 cm, PGR > 50%, histological grade <3, Ki-67 < 20%) characterize subpopulations with low CPRS values which correspond to a lower therapeutic benefit. Conversely, the efficacy of the dose-dense chemotherapy appears to be greater for subpopulations with high CPRS values where variables such as a number of lymph nodes ≥4, a tumor size >2 cm, and Ki-67 levels ≥20% are mostly concentrated.

The subsequent DFS-oriented STEPP analysis showed, moreover, an increasingly higher benefit for the dose-dense regimen across medium–high-risk patients, with advantage mitigation for the highest risk subgroup. On the other hand, the absolute benefit was consistently higher in the third and the fourth CPRS quartile (respectively, NNT 5 and 6) (Fig. 3c), compared to the first CPRS quartile (NNT 154), a trend that was also confirmed in terms of 5-year and 9-year DFS.

These apparently counterintuitive results could be due to a lower relative benefit linked to the specific tumor biology of the fourth CPRS quartile which is undifferentiated and therefore potentially less chemosensitive. On the other hand, the higher clinical risk of this subgroup amplifies small relative benefits in higher absolute benefits.

The use of dose-dense chemotherapy in patients with hormone receptor-positive has been largely debated. It is generally believed that hormone receptor-positive tumors are intrinsically less responsive to chemotherapy than hormone receptor-negative tumors. In the CALGB combined analysis, the absolute benefit due to the dose-dense schedule was greater for patients with hormone receptor-negative disease compared with that observed for patients with hormone receptor-positive disease^10^. The authors have provided some possible explanations for these observations. One hypothesis considered the fact that, although the chemotherapy regimen was randomly assigned across treatment arms, endocrine treatment was not. Different agents and treatment compliances could randomly affect overall efficacy and consequently alter the final conclusions. Furthermore, the risk reduction that may be achieved with chemotherapy is difficult to detect in the first years after treatment when the risk of relapse for luminal subtypes is lower than in hormone receptor-negative subgroups. Regardless, the population of hormone receptor-positive could be heterogeneous with respect to intrinsic sensitivity to chemotherapy.

Several studies have proposed gene expression analysis as a means to define the benefit of chemotherapy in patients with hormone receptor-positive HER2-negative breast cancer^12^.

Interestingly, an interim analysis (median follow-up: 5.1 years) of phase 3 RxPONDER trial, presented at the San Antonio Breast Cancer Symposium 2020, showed that adding chemotherapy to endocrine therapy did not improve outcomes for postmenopausal women with early-stage, node-positive, luminal breast cancer in comparison to endocrine therapy alone. Specifically, these findings relate to postmenopausal patients with involvement of 1–3 lymph nodes and defined as low risk on the basis of recurrence score ≤25 on the 21-tumor gene expression assay (Oncotype Dx). On the other hand, women with the same characteristics but premenopausal were found to have benefited from chemotherapy, probably as a result of the endocrine effect of chemotherapy-induced ovarian suppression^13^.

Although gene expression analysis was not performed in patients of the GIM2 trial, it seems reasonable to assume that the estimation of the benefit from adding chemotherapy to endocrine therapy in low risk (i.e., recurrence score ≤25) patients also applies to dose-dense chemotherapy. Of note, a pooled analysis of two randomized clinical trials that evaluated the efficacy of adjuvant dose-dense chemotherapy in premenopausal breast cancer patients did not show an increased risk of treatment-induced amenorrhea with dose-dense chemotherapy^14^. Therefore, in premenopausal patients, it is difficult to hypothesize a greater chemo-induced endocrine effect of dose-dense chemotherapy compared to standard-interval chemotherapy.

The Early Breast Cancer Trialists’ Collaborative Group (EBCTCG) patient-level meta-analysis further dissected the role of dose-dense adjuvant regimens in eBC^15^. The pooled data of 37,298 women enrolled in 26 different randomized trials were analyzed, with recurrence and breast cancer mortality as primary outcomes. In the total population, dose-intense strategies were associated with a reduction of both recurrences (RR 0.86; 95% CI 0.82–0.89; *P* < 0.0001) and breast cancer mortality (RR 0.86; 95% CI 0.77–0.96). Recurrence reduction was similar in hormone receptor-negative and hormone receptor-positive eBC patients (RR 0.82; 95% CI 0.71–0.95 and RR 0.83, 95% CI 0.75–0.93, respectively), while a slightly different delta was highlighted in terms of 10-year recurrence (3.7% vs 3.1% in hormone receptor-negative and hormone receptor-positive eBC patients, respectively), consistently with the assumption that, at the same level of relative risk reduction, higher-risk groups experience a greater absolute benefit.

In fact, while acknowledging the value of the EBCTG meta-analysis, we conducted an exploratory analysis of the GIM2 study in order to understand granularly how the different variables contribute to the risk of relapse and the consequent estimate of the therapeutic benefit based on that risk.

With a median follow-up of 7 years, GIM2 is the dose-dense focused trial with the longest follow-up and is therefore the best candidate to better explore tailored treatment strategies in hormone receptor-positive eBC. By combining well-established prognostic factors in a CPRS, the present analysis was capable to represent risk as a continuous variable and to identify and describe specific risk subgroups that could have a differential benefit from dose-dense chemotherapy. In particular, the CPRS quartile 4 is characterized by specific biological characteristics that confer a potentially chemosensitive phenotype and are typically associated with a luminal B-like profile, such as low ER and PGR levels and high KI67. Notably, the association with younger age could have intriguing biological implications^16^. It has been previously reported that breast cancer of the young, particularly the hormone receptor-positive subtype, is a peculiar biological entity that can differ from the older counterpart in several molecular aspects and in particular through the enrichment of biological processes related to stemness and growth factor signaling and the downregulation of apoptosis-related genes^17^. These aspects could deeply affect the tumors’ response to chemotherapy which could justify the need for a more dose-intense treatment schedule^14^.

Dose-dense adjuvant chemotherapy showed a consistent benefit in node-positive eBC patients with hormone receptor-positive HER2-negative disease, but its effect varied according to CPRS. Therefore, dose-dense adjuvant chemotherapy should be taken into consideration also in the hormone receptor-positive subgroup. This study offers a potential hint towards a tailored approach based on broader multiparametric criteria, capable of capturing the wide and granular risk spectrum of hormone receptor-positive eBC.

## Methods

### Cohort design

The GIM2 is a multicenter, randomized phase 3 trial, with a 2 × 2 factorial design aiming to address both the role of the addition of fluorouracil to epirubicin, cyclophosphamide, and paclitaxel and the role of the dose-dense schedule in the adjuvant treatment of patients with node-positive eBC^11^.

The study population included 2198 patients from 81 Italian centers. The primary endpoint was DFS, defined as the time from random assignment to local or distant recurrence, contralateral or ipsilateral breast tumor (excluding ductal carcinoma in situ), second primary malignancy, death from any cause, and loss to follow-up or end of the study, whichever came first.

As a sub-analysis of the GIM2 randomized multicenter clinical trial, the study was approved by ethics committees of all participating institutions. Written informed consent was obtained from all patients before study entry. Participating centers and principal investigators are listed in Supplementary Data 1.

### Composite risk score analysis

The present analysis examined the absolute treatment effect through a composite measure of recurrence risk to better individualize the clinical algorithm in the choice between dose-dense and standard-interval chemotherapy in patients with hormone receptor-positive HER2-negative eBC (*N* = 1131).

Patients with hormone receptor-positive HER2-positive eBC were excluded from the current analysis to avoid the potential confounding effect of trastuzumab use in only a minority of these patients and the differential observed benefit of dose-dense chemotherapy in this subgroup^18^.

Applying to hormone receptor-positive HER2-negative patients the Cox proportional hazards model, the risk of recurrence (hazard ratio) related to each prognostic factor of the patients was estimated, such as age (<35, 35–39, 40–44, 45–49, ≥50 years), number of positive nodes (1–3, ≥4), tumor size (≤2, >2 cm), estrogen receptor (ER, < 50%, ≥50%), progesterone receptor (PGR, < 20%, 20–49%, ≥50%), histological grade (1, 2, 3), KI67 ( < 14%, 14–19%, 20–25%, ≥26%). The groupings of prognostic factors followed the usual clinical cut-points as defined by the St Gallen Consensus statements with the exception of estrogen receptor due to the low number of patients with values less than 10% (*n* = 31) and KI67 where a high proportion of patients (*n* = 294) showed values equal to or greater than 26^19^. Multiple Imputation procedure was applied to handle missing values^20,21^. The CPRS was then computed, for each patient, through the equation CPRS = ***β***_**1**_***X***_**1**_ + … + ***β***_**n**_***X***_**n**_ (1). In Eq. (1) for each prognostic factor, ***β***_**i**_ is the logs (hazard ratio) and ***X***_**i**_ is an indicator variable taking value 1 if the factor is present and 0 otherwise. Thus, the CPRS was estimated as the sum of the logs (hazard ratio) of the prognostic factors of each patient. We explored the treatment effect compared to the values of CPRS by using the Sub-population Treatment Effect Pattern Plot (STEPP) process^21^. Proportional hazards assumption was satisfied before and after multiple imputation^22^. The number needed to treat (NNT) was computed as the reciprocal of the difference between the absolute risks of failure in the dose-dense and standard therapy arms, respectively.

Patients were ordered from the lowest to the highest value of CPRS. Two quantities were chosen: N1, the size of the cohorts, and N2 the maximum number of patients shared by two cohorts where N1 > N2. The process began with the cohort of first N1 patients in the ordered list. At the next step, the N2 patients with the highest values of the CPRS in the previous cohort were moved to a new cohort in addition to the N1–N2 patients that followed in the ordered list. The process ended when all patients were included in at least one cohort. In this analysis, it was chosen N1 = 300 and N2 = 250. For each cohort, the HR relative to treatment was estimated. The heterogeneity of HRs along the cohorts defined by STEPP analysis was tested by means of permutation test^23^.

Statistical analysis was performed using R (The R Foundation for Statistical Computing. version 3.3.1 (2016-06-21)) and STATA (StataCorp. (2015) Stata Statistical Software: Release 14.2. College Station, TX: StataCorp LP) software packages.

### Reporting summary

Further information on research design is available in the Nature Research Reporting Summary linked to this article.

## Supplementary information

Supplementary Data 1

Reporting Summary

## Data Availability

The data generated and analyzed during this study are described in the following data record: 10.6084/m9.figshare.14547321^24^. The data are contained in two Excel spreadsheets: “Patient survival data.xlsx” and “Patient baseline, tumour and treatment data.xlsx”. These spreadsheets are housed on institutional storage and are not publicly available for the following reason: data contain information that could compromise research participant privacy. However, the data can be made available upon reasonable request to the corresponding author. The dataset analyzed during this study is described with more details in the following manuscript: 10.1016/S0140-6736(14)62048-1^11^.
