# Supplementary material for: Composite risk and benefit from adjuvant dose-dense chemotherapy in hormone receptor-positive breast cancer
Source: NPJ Breast Cancer. 2021 Jun 28;7:82. doi: 10.1038/s41523-021-00286-w (PMC8238951; doi:10.1038/s41523-021-00286-w)
Supplement: Supplementary file 1 — Supplementary Data 1 [file 41523_2021_286_MOESM1_ESM.pdf]

M. Venturini, A. Abate, S. Pastorino, G. Canavese, C. Vecchio, M. Guenzi, M. Lambertini, A. Levaggi, S. Giraudi(Genova); V. Accortanzo (Torino); C. A. Floris (Cagliari); E. Aitini (Mantova); G. Fornari (Torino); S. Miraglia (Torino); G. Buonfanti (Napoli); M. C. Cherchi (Cagliari); F. Petrelli (Treviglio); A. Vaccaro (Frosinone); E. Magnolfi (Sora); A. Contu (Sassari); R. Labianca (Bergamo); A. Parisi (Roma); C. Basurto (Perugia); F. Cappuzzo (Livorno); M. Merlano (Cuneo); S. Russo (Udine); M. Mansutti (Udine); E. Poletto (Udine); M. Nardi (Reggio Calabria); D. Grasso (Pavia); A. Fontana (Pisa); L. Isa (Gorgonzola); M. Comandè (Gergonzola); L. Cavanna (Piacenza); S. Iacobelli (Chieti); S. Milani (Trieste); G. Mustacchi (Trieste); S. Venturini (Massa); A. F. Scinto (Roma); M. G. Sarobba (Sassari); P. Pugliese (Como); A. Bernardo (Pavia); I. Pavese (Roma); M. Coccaro (Rionero in Vulture); B. Massidda (Monserrato); M.T. Ionta (Monserrato); A. Nuzzo (Lanciano); L. Laudadio (Lanciano); V. Chiantera (Cambobasso); R. Dottori (Grosseto); M. Barduagni (Albano Laziale); F. Castiglione (Alba); F. Ciardiello (Napoli); V. Tinessa (Benevento); A. Ficorella (L'Aquila); L. Moscetti (Viterbo); I. Vallini (Varese); G. Giardina (Varese); R. Silva (Fabriano); M. Montedoro (Città di Castello); E. Seles (Biella); F. Morano (Lugo); G. Cruciani (Lugo); V. Adamo (Messina); A. Pancotti (Sant'Omero); V. Palmisani (Palermo); A. Ruggeri (Pozzuoli); E. Cammilluzzi (Roma); F. Carrozza (Campobasso); M. D'Aprile (Latina); M. Brunetti (Albano Laziale); P. Gallotti (Vigevano); E. Chiesa (Vigevano); F. Testore (Asti); A. D'Arco (Pagani); A. Ferro (Trento); A. Jirillo (Padova); M. Pezzoli (Ancona); G. Scambia (Roma); C. Iacono (Ragusa); P. Masullo (Vallo della Lucania); G. Tomasello (Cremona); G. Gandini (Correggio); A. Zoboli (Correggio); C. Bottero (Alessandria); M. Cazzaniga (Monza); G. Genua (Ariano Irpino); S. Palazzo (Cosenza); M. D'Amico (Genova); D. Perrone (Saluzzo) – all in Italy

Supplementary Data 1. Participating centers and principal investigators
