# Supplementary material for: Composite risk and benefit from adjuvant dose-dense chemotherapy in hormone receptor-positive breast cancer
Source: NPJ Breast Cancer. 2021 Jun 28;7:82. doi: 10.1038/s41523-021-00286-w (PMC8238951; doi:10.1038/s41523-021-00286-w)
Supplement: Supplementary file 2 — Reporting Summary [file 41523_2021_286_MOESM2_ESM.pdf]

nature research

Corresponding author(s): Fabio Puglisi, MD, PhD

Last updated by author(s): Jan 8, 2021

Reporting Summary

Nature Research wishes to improve the reproducibility of the work that we publish. This form provides structure for consistency and transparency in reporting. For further information on Nature Research policies, see our Editorial Policies and the Editorial Policy Checklist.

Statistics

For all statistical analyses, confirm that the following items are present in the figure legend, table legend, main text, or Methods section.

n/a	Confirmed	
☐	☒	The exact sample size (n) for each experimental group/condition, given as a discrete number and unit of measurement
☒	☐	A statement on whether measurements were taken from distinct samples or whether the same sample was measured repeatedly
☐	☒	The statistical test(s) used AND whether they are one- or two-sided<br>*Only common tests should be described solely by name; describe more complex techniques in the Methods section.*
☐	☒	A description of all covariates tested
☐	☒	A description of any assumptions or corrections, such as tests of normality and adjustment for multiple comparisons
☐	☒	A full description of the statistical parameters including central tendency (e.g. means) or other basic estimates (e.g. regression coefficient) AND variation (e.g. standard deviation) or associated estimates of uncertainty (e.g. confidence intervals)
☐	☒	For null hypothesis testing, the test statistic (e.g. F, t, r) with confidence intervals, effect sizes, degrees of freedom and P value noted<br>*Give P values as exact values whenever suitable.*
☒	☐	For Bayesian analysis, information on the choice of priors and Markov chain Monte Carlo settings
☒	☐	For hierarchical and complex designs, identification of the appropriate level for tests and full reporting of outcomes
☐	☒	Estimates of effect sizes (e.g. Cohen's d, Pearson's r), indicating how they were calculated

Our web collection on statistics for biologists contains articles on many of the points above.

Software and code

Policy information about availability of computer code

Data collection	Data collection was performed through the OncoTech eCRF online platform
Data analysis	Statistical analysis was performed using R (The R foundation for Statistical Computing. version 3.3.1 (2016-06-21)) and STATA (StataCorp. (2015) Stata Statistical Software: Release 14.2. College Station, TX: StataCorp LP) software packages.

For manuscripts utilizing custom algorithms or software that are central to the research but not yet described in published literature, software must be made available to editors and reviewers. We strongly encourage code deposition in a community repository (e.g. GitHub). See the Nature Research guidelines for submitting code & software for further information.

Data

Policy information about availability of data

All manuscripts must include a data availability statement. This statement should provide the following information, where applicable:

- Accession codes, unique identifiers, or web links for publicly available datasets
- A list of figures that have associated raw data
- A description of any restrictions on data availability

Data from patients involved in the GIM2 trial are not publicly available to protect patient privacy but are available from the corresponding author on reasonable request.

Field-specific reporting

Please select the one below that is the best fit for your research. If you are not sure, read the appropriate sections before making your selection.

☒ Life sciences ☐ Behavioural & social sciences ☐ Ecological, evolutionary & environmental sciences

For a reference copy of the document with all sections, see nature.com/documents/nr-reporting-summary-flat.pdf

Life sciences study design

All studies must disclose on these points even when the disclosure is negative.

Sample size	Based on a 2x2 factorial design and the primary endpoint Disease Free Surviva, the sample size was 2091 patients in the master protocol. The study was then focused on ER/PR positive EBC patients
Data exclusions	No data were excluded
Replication	N/A
Randomization	Eligible patients were randomly allocated in a 1:1:1:1 ratio with a centralised, interactive online system to receive either dose-dense chemotherapy or to standard-interval chemotherapy
Blinding	The study was an open-label, phase 3 trial

Reporting for specific materials, systems and methods

We require information from authors about some types of materials, experimental systems and methods used in many studies. Here, indicate whether each material, system or method listed is relevant to your study. If you are not sure if a list item applies to your research, read the appropriate section before selecting a response.

Materials & experimental systems

n/a	Involved in the study	
☒	☐	Antibodies
☒	☐	Eukaryotic cell lines
☒	☐	Palaeontology and archaeology
☒	☐	Animals and other organisms
☐	☒	Human research participants
☐	☒	Clinical data
☒	☐	Dual use research of concern

Methods

n/a	Involved in the study	
☒	☐	ChIP-seq
☒	☐	Flow cytometry
☒	☐	MRI-based neuroimaging

Human research participants

Policy information about studies involving human research participants

Population characteristics	Women with histologically proven unilateral operable invasive breast cancer confined to the breast and ipsilateral axilla were enrolled, permitted age went from 18 to 70 years, Eastern Cooperative Oncology Group performance status had to be 1 or lower
Recruitment	Patients were recruited across 81 Italian Cancer centers according to the study protocol. Randomization was then applied.
Ethics oversight	The study was approved by the Ethics Committee of all participating sites

Note that full information on the approval of the study protocol must also be provided in the manuscript.

Clinical data

Policy information about clinical studies

All manuscripts should comply with the ICMJE guidelines for publication of clinical research and a completed CONSORT checklist must be included with all submissions.

Clinical trial registration	NCT00433420
Study protocol	http://www.oncotech.org/gim2
Data collection	Data were collected at baseline and at each clinical evaluation through the centralized Oncotech online eCRF platform.

Outcomes

The primary study endpoint was disease- free survival; secondary endpoints included overall survival and safety. Disease-free survival was computed from the date of randomisation to the date of local recurrence, distant metastases, contralateral or ipsilateral breast tumour (excluding ductal carcinoma in situ), second primary malignancy, death from any cause, and loss to follow-up or end of study, whichever came first. Disease-free survival times of patients without a disease-free survival event when lost to follow-up or at the time of study closure were censored on the date of the last contact. The defi nition of disease-free survival corresponds to the Invasive Disease Free Survival defi ned by Hudis and colleagues.
Similarly, overall survival was computed from the day of randomisation to the date of death from any cause, loss to follow-up, or the end of study. Adverse events were assessed clinically and by haematological and biochemical measurements throughout chemotherapy. Adverse events were graded according to the National Cancer Institute common toxicity.
